# Some Biological Aspects of Bigeye Scad, *Selar crumenophthalmus* from Bangaa Faru, Maldives

**DOI:** 10.21315/tlsr2017.28.2.10

**Published:** 2017-07-31

**Authors:** Nik Fadzly, Shaza Adeeb, Amir Shah Ruddin Md Sah

**Affiliations:** 1 School of Biological Sciences, Universiti Sains Malaysia, 11800 USM Pulau Pinang, Malaysia; 2 School of Life Sciences, Huxley Building, Keele University, Keele, Staffordshire, ST5 5BG. United Kingdom

**Keywords:** *Selar crumenophthalmus*, Gonado-somatic Index, Reproductive Biology, Male’ Maldives, Size at Maturity

## Abstract

In this paper, we studied some biological aspects of big eye scad (*Selar crumenophthalmus*) from Maldives. The fish sample was collected from the local fish market at Bangaa Faru, Male, Maldives. The length of the samples were ranged from 7.7 cm to 24.5 cm (mean value = 16.85 ± 2.82 cm) in fork length. Body weight ranged between 8 g to 255.6 g (mean value = 87.76 ± 40.41 g). The exponent values (b slope) of lengthweight relationship of *S. crumenophthalmus* are 2.9838 for females and 2.7687 for males; indicating negative allometric growth pattern for both sexes. Synchronous reproductive behaviour was observed in both sexes and a pronounced peak of Gonadosomatic index was observed in females in January 2013. It is estimated that length at first maturity (L_50_) for females is at 19.39 cm FL and for males at 21.76 cm FL. Our result also suggest that big eye scad have a reduced swimming capability, resulting the species to be easily caught. Careful planning and management should be implemented to prevent the big eye scad from being overfished.

## Abstract (ru)

Dalam makalah ini, kami mengkaji beberapa aspek biologi daripada *big eye scad* (*Selar crumenophthalmus*) dari Maldives. Sampel ikan dikumpulkan dari pasar ikan tempatan di Bangaa Faru, Male, Maldives. Panjang sampel adalah antara 7.7 cm hingga 24.5 cm (nilai min = 16.85±2.82 cm) mengikut panjang cabang sirip (FL). Berat badan antara 8 g hingga 255.6 g (nilai min = 87.76±40.41 g). Nilai eksponen (cerun b) hubungan berat badan *S. crumenophthalmus* adalah 2.9838 untuk betina dan 2.7687 untuk jantan; menunjukkan corak pertumbuhan alometrik negatif bagi kedua-dua jantina. Tingkah laku pembiakan selari diperhatikan dalam kedua-dua jenis jantina dan puncak indeks Gonadosomatic yang diperhatikan pada ikan betina pada bulan Januari 2013. Dianggarkan bahawa panjang pada kematangan pertama (L50) bagi ikan betina adalah pada 19.39 cm FL dan bagi ikan jantan pada 21.76 cm FL. Hasil kajian juga mencadangkan bahawa *big eye scad* mempunyai keupayaan renang berkurangan, menyebabkan spesies mudah ditangkap. Perancangan dan pengurusan yang teliti perlu dilaksanakan untuk mengelakkan masalah mata yang terlalu besar.

## INTRODUCTION

Bigeye scad (*Selar crumenophthalmus)* of the family Carangidae is a small coastal pelagic fish that is abundantly found in the coastal waters of the Maldives. It is quite a popular fish. Other than being consumed as a traditional delicacy, the fish are also used as bait to catch tuna. Therefore, bigeye scad is considered both socially and economically important for the Maldives.

**Figure 1:**
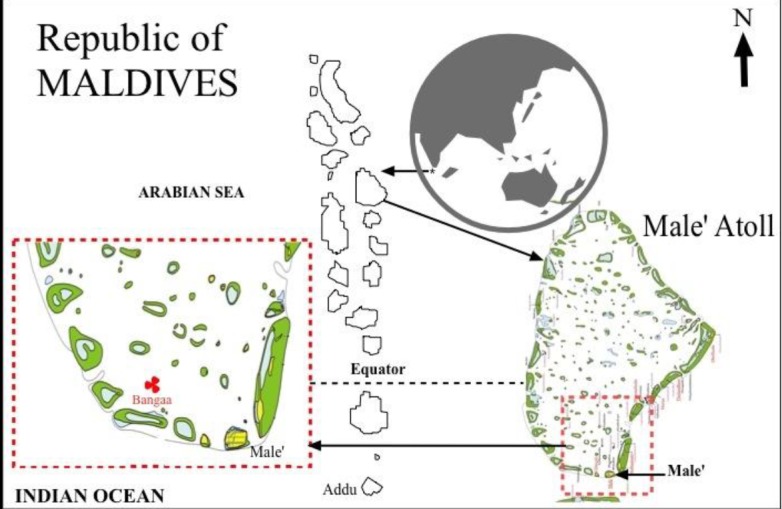
Location of Bangaa Faru sampling site.

Maldives is a small island nation comprising of 1200 coral islands grouped into 19 widely dispersed atolls covering an area of nearly 90 000 km^2^ in the Indian Ocean (Adam 2006) (Fig. 1). Although the country’s Exclusive Economic Zone (EEZ) covers an area of 1 million km^2^, the main capital, Male is only 2 km in length and 1 km in width with a population of 109,494 (HIES 2012). As a nation surrounded by water, fishing is the most dominant activity and the second largest contributor to the country’s GDP after tourism industry (Latheefa 2004; Solah 2007; Charles 2005; Latheefa *et al.* 2011).

To maintain an efficient and sustainable fishing industry, one of the main aspects that needs to be monitored is the understanding of the fish reproductive biology. Information about the reproductive biology could be conveyed to the related authorities to monitor the fish productivity and prevent over-exploitation of the fish population (Morgan 2008). Such information also suggests the suitability of fish as biological indicators of environmental stability (John *et al.* 2009).

Sex ration provides the basic information needed for the assessment of the potential of reproduction and stock size estimation (Vicentini & Araujo 2003). Another important is the correct estimations of size of first maturity or length at which 50% of the fish are mature are essential in fish stock management (Nelson *et al.* 2009). The study of length weight relationship in fishes is used in various applications (Beverton & Holt 1957; Ricker 1958) such as the estimation of stock size and exploitation (Dulčič & Kraljević 1996). The general expectation for length weight relationship is that, the variation of weight does not follow the cube law of length-weight relationship (Rounsefell & Everhart 1953), since fishes normally do not retain the same physiology throughout their lifespan (Le Cren 1951).

With the exception of Hafiz (1990), there is no detailed analysis of the biological analysis of *S. crumenopthalmus* from the Maldivian waters. There are other studies from all over the world, Kawamoto (1973) studied a detailed biology of the fish, followed by Clarke and Privitera (1995) in Hawaii. A growth length frequency analysis was conducted in Indonesia by Sadhamoto and Atmadja (1985). A more modern approach using ELEFAN in FISAT software was conducted by Mansor *et al.* (1996) for bigeye scad in Malaysia. According to Adeeb *et al*. (2014), based on the exploitation rate, the fish is dangerously overexploited in Bangaa Faru, in Male’ atoll Maldives. Alarmingly Panda *et al.* (2015) also shows a similar trend of overexploitation in Mumbai, north-west coast of India.

The objectives of this study are to discover sex ratio, classify the gonad stages, calculate the gonadosomatic index and to estimate the length at first maturity and length weight relationship for bigeye scad *S. crumenophthalmus* in Bangaa Faru, Maldives.

## MATERIALS AND METHODS

### Study Site and Sampling Procedure

A total of 1648 bigeye scads (356 males, 143 females and 1149 immature individuals) was randomly sampled from the commercial catch in the local fish market in Male, from September 2012 to February 2013. The samples were caught from Bangaa Faru area, a popular bigeye scad fishing spot located near Male. The samples were fresh captured fish. Measurements and dissections were quickly performed to prevent measurement bias and sample decay. The fishes were sampled from various vendors to reduce selective preference bias.

### Length and Weight Measurement

Each individual fish was measured for the fork length (FL) to the nearest centimetre using a tape measure and body weight (BW) was recorded for nearest 0.1 g using a portable digital scale (HD005 electronic scale).

### Gonad Extraction and Measurements

Each fish was dissected in the abdomen region using scissors and knife. The gonads were usually located below the intestine and near the backbone base. Gonad weight was taken to the nearest 0.1 g using an electronic balance (model HD005) and length of the gonads were measured on a measuring board using a ruler to the nearest centimetre.

### Sex Ratio

The sex of each specimen was identified by physical examination of the gonads. The proportion of the two sexes relative to one another was used to calculate the sex ratio.

### Gonad Stages Identification

Macroscopic identification of gonads was done based on Five – point Maturity scales for partial spawners (Holden & Raitt, 1974). The gonad developmental stages are categorised as immature (Ovaries and testis about 1/3 length of the body cavity), maturing (Ovary and testis about ½ length of the body cavity and ovary are pinkish without visible ova to the naked eye and whitish testis), ripening (Ovary and testis takes about 2/3 length of the body cavity and ovary with granular appearance and whitish to creamy testis), ripe (Ovary and testis from 2/3 to the full length of the body cavity. Ovary with conspicuous superficial blood vessels and testis is whitish to creamy and soft) and last stage as spent (Ovary and testis shrunken to about half-length of the body cavity and loose walls).

### Gonadosomatic Index (GSI)

The gonadosomatic index was calculated as a percentage of body mass. It is represented by the formula: GSI = [Gonad Weight / Total Tissue Weight (weight of fish)] x 100. This was calculated for each individual and a monthly average for each sex was established. The GSI calculation were pooled based on the sex of fish regardless of the gonad maturity stage.

### Length at First Maturity (L_50_)

Length at first maturity or size in which 50% of the individuals are mature was calculated using the following equation:

The L_50_ was estimated using the following equation (Silberberg *et al.* 2001):

$$\text{P}=\frac{1}{1+e^{-a(FL-b)}}$$

where *P* = proportion of mature fish at a specific length class (measured as total length); *a* and *b* are model parameters to be estimated; FL = fork length.

### Length Weight Relationship

The relationship between the fork length and weight of the fish were estimated by:

$$\text{W}=\text{a }{\text{L}}^{b}$$

W is weight (g), L is fork length (cm), *a* is constant of proportionality and *b* is the length of exponent or slope. The values of the exponent *b* provide information on the growth of the fish. When *b* is equal to three (*b* = 3), increase in weight is isometric. When the value of *b* is other than 3, weight increase is allometric, (positive allometric if *b* > 3, negative allometric if *b* < 3). Model parameters for L_50_ and length-weight relationship were obtained using calculation on SPSS 21 and Microsoft Excel.

## RESULTS

### Sex Ratio

**Figure 2:**
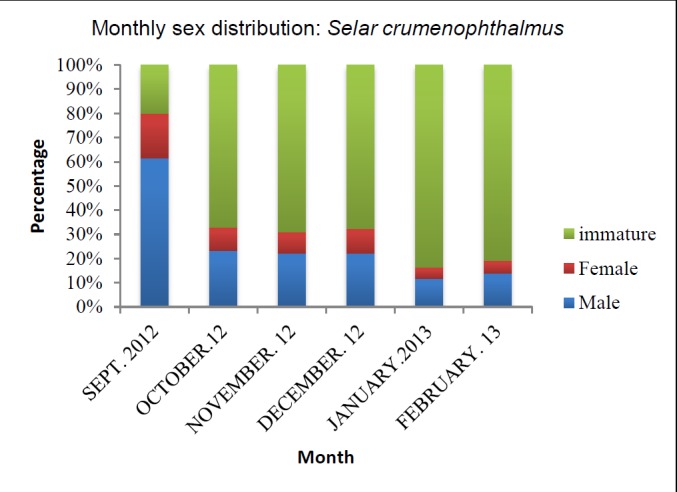
Monthly sex distribution of *Selar crumenophthalmus*.

Out of 1648 bigeye scads collected, 69.7% of the specimens were immature, while 21.6% were males and 8.7% were females (Table 1). Hence the male female sex ratio is 1:0.39. The male to female ratio was low in September (1:0.30) and highest in December (1:0.46). Except during September 2012, the number of indeterminate or immature individuals was high during the study period. The number gradually increased until December 2013 then the numbers decreased in February 2013 (January 2013 = 365 immature individuals to February 2013 = 64 immature individuals). Figure 2 shows the percentage of males, females and immature individuals.

Gonad maturity stages identification: The ovaries were orange in colour, thin and ova were not visible to the naked eye during gonad stage one. Colour, size and ova visibility gradually increased as the ovaries matured. Ovaries of gonad stage four appeared bright orange in colour with conspicuous superficial blood vessels and ripe ova were visible to the naked eye and occupied almost the entire body cavity (Fig. 3a). The immature testes were flatter and pale white. As the testes matured, it became more whitish in colour and broader and occupied about three quarters of the body cavity (Fig. 3b). The weight of the gonads in males and females increased as they matured. Sexual dichromatism was observed in the mature fish. The soft portion of the anal fin appeared to be black in males and white in females.

Gonad stages identification was done for four months, starting from November 2012 to February 2013. Majority of observed male gonads were at stage one and this number declined as the study progressed. Except for December 2013, gonad stage five was not observed at any time (Fig. 4a). In this study fully matured gonads were seldom observed. However, a steady increase in the occurrence of gonad stages II and III was observed.

In females, observation of gonad maturity stage I gradually decreased from 17 individuals having gonad stage I in November to one individual in February (Fig. 4b). Similar to males, females also had an increase in the occurrence of gonad stages from stage I to stage IV and then the number declined from January 2013 onwards. Unlike male individuals, in females all the gonadal stages were observed more often. However, gonad stage five was not observed in females. The maximum-recorded weight of the gonads was 10.7 g (mean value = 1.83±1.53) for a stage four female gonad and the maximum-recorded length was 6.1 cm (mean value = 2.14±1.89).

**Figure 5:**
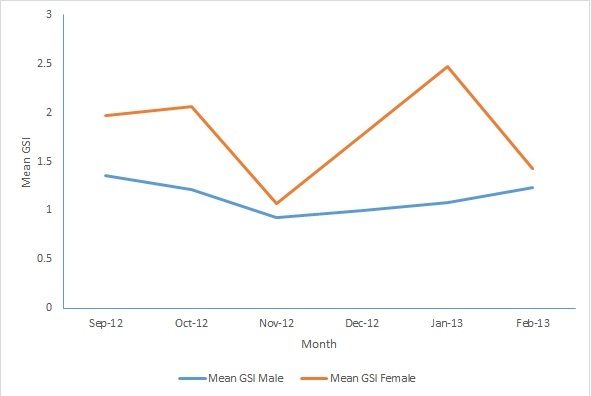
Monthly GSI of *Selar crumenophthalmus.*

Gonadosomatic index: The mean GSI value increased to 2.06 in October 2012 from 1.97 in September 2012 and then the value plunged to 1.07 in November 2012. Afterwards, mean GSI value rose to its peak value of 2.47 in January 2013 and later decreased to 1.43 in February 2013. In males, the mean GSI value gradually decreased from 1.36 in September to 0.93 in November. Subsequently, it steadily increased from 1.00 in December to 1.23 in February 2013. Unlike female bigeye scads, male GSI value does not show a distinctive peak. However, there were considerable differences between the GSI values of males and females. The index value of females was higher than of males and the variation of gonad index corroborates the observations of maturity stages (Fig. 5).

Length at first maturity: The smallest female observed was 17.4 cm FL whereas smallest male observed was 15.5 cm FL. The smallest mature male and female observed have a fork length of 17.5 cm and 18.5 cm respectively. It was found that fish lengths smaller than 15.0 cm FL have been always immature and fish lengths above 19.5 cm FL were mature throughout the study sample. It is estimated that *S. crumenophthalmus* from Bangaa Faru attains length at first maturity for females at 19.39 cm FL whereas for males at 21.76 cm FL (Fig. 6a and 6b respectively). The fork length of the combined study sample ranged from 7.7 cm FL to 24.5 cm FL (mean value = 16.85±2.82 cm).

Length weight relationship: Overall 1648 specimens were chosen for the study of length weight relationship. There was a significant relationship between the fork length (t = 248.375, df = 355, P < 0.0001) and weight (t = 78.134, df = 355, P < 0.0001) of males and females of *Selar crumenophthalmus* in the Bangaa faru. The *b* values of *S. crumenophthalmus* are 2.9838 for females (Fig. 7a), 2.7687 for males (Fig. 7b) and 2.8303 for pooled data (Fig. 7c). The results indicate that the fish follow the cube law. The growth is proportionally three-dimensional. Based on the slope values of *b,* all the categories fall into the lighter group (*b* < 3). Hence, *S. crumenophthalmus* in Bangaa faru shows negative allometric growth. The body weight of the sample ranged from 7.7 g to 255.6 g (mean value = 87.76±40.41 g). The mean weight for males is 121.50±29.34g, female is 145.22±33.07 g and indeterminate or immatures have a mean weight of 70±29.50 g). The minimum and maximum fork length for females are 17.4 cm and 24.5 cm (mean value = 20.19±1.55) respectively. For males the minimum and maximum fork length are 14.7 cm and 23.6 cm (mean value = 19.04±1.51) respectively.

## DISCUSSION

*Selar crumenophthalmus* is widely distributed in the warm coastal waters of the Atlantic, Indian and Pacific oceans (Kazama 1977; Mablouke *et al.* 2013). The fish is heterosexual and iteroparous and aggregate to spawn (Clarke & Privitera 1995; Weng & Sibert 2000). The species exhibits sexual dimorphism and sexual dichromatism (Shameen & Dutt 1984; Clarke & Privitera 1995;).

Previous study from Kawamoto (1973) suggests that the bigeye scad reproduction extends over a period of six to seven months between March and September. However, based on our results, the appearances of immatures were higher in October to February suggests a slight shorter recruitment period in

Bangaa faaru. Our result also suggests the peak spawning period for the females is in January based on the highest GSI value. We acknowledge that our data collection did not encompass a full one-year observation due to time and budget limitation. However, we are confident that based on the quantity of samples, our data could provide the necessary information. Clarke and Privitera (1995) reported that bigeye scad in Hawaiian waters spawns every three days during their spawning season from April to October. Mansor *et al.* (1996) reported the spawning of bigeye scads in the East coast of Peninsular Malaysia using the mean GSI and suggests that spawning is from April–May and November–December in 1993 and February–March and August–October in 1994. Roux and Conand (2000) stated that *S. crumenophthalmus* have an annual reproductive cycle and spawning occurs mostly from October to December. Iwai *et al.* (1996) observed that natural spawning of a captive sample from Hawaiian waters occurred during their first year of captivity and the brood stock spawned repetitively throughout the second and third year in culture. Spawning in captive fish occurred during nighttime with a majority of spawning activity during the predawn hours (Iwai *et al.* 1996) whereas Podosinnikov (1990) reported that mass spawning occurred during nighttime in Gulf Aden.

Roos *et al.* (2007) noted that, monthly distribution of males and undetermined or immatures occurred in November, gradually attaining adulthood from December to February. A similar trend also was observed in this study as the number of immatures were higher in the study period of six months and the maximum percentage of mature individuals were observed in September and February. Clarke and Privitera (1995), based on their sample from Hawaii; had noted that the smallest mature male was 19.9 cm in standard length for males whereas for females the standard length was more than 21.0 cm. Kawamoto (1973) reported that length at first maturity for Hawaiian bigeye scad as 23.0 cm and in Indian waters bigeye scad size ranges between 10 cm in 6 months old fish and 26.5 cm in 3 years old fish (Joseph & Jayaprakash 2003). However, in this study, the smallest individual observed was 84.0 cm (in FL) and the largest was 245 cm (in FL). In the Philippines the maximum length observed was 23.0 cm FL (Dalzella & Peñaflor 1989) and 22 cm FL was observed on Reunion Island (Roos *et al.* 2007).

The findings of the growth this study was similarly demonstrated by Gonzales *et al.* (2000) which also it showed a negative allometry (*b* < 3) of 2.78. However, the findings of Roos *et al.* (2007) and Rumpet *et al.* (1997) differ from this study, as they showed that *S. crumenophthalmus* has a positive allometry (3.25). Our result suggests that the fish grows faster in weight than length (Froese 2006; Lleonart *et al*. 2000). Differences in length weight relationships can occur due to environmental, seasonal changes and population (Froese 2006). Roos *et al.* (2007) and Rumpet *et al.* (1997) reported that the specimens were caught by hand line and beach seine for the former study and a high opening otter trawl net for the latter study. For this study, the fishing gear used was pole and line and this could have created a bias in the results (Kipling 1962). In this study, the samples were obtained from the commercial market, which infers that most of the stock are the most easily and commonly caught among the fishermen. The reduced in length whilst an increase in body weight do suggest that the bigeye scad population in Bangaa Faaru have a reduced swimming capability, hence why it is quite easily fished. While this is beneficial to the local fishermen, such allometric trend could lead to over-exploitation of the bigeye scad population. Such predicament had been reported by Adeeb *et al.* (2014) and Panda *et al.* (2015)

This study provides the first description of *S. crumenophthalmus* reproduction in Maldivian waters. Our overall results provide a preliminary outlook on the reproductive biology of bigeye scad in Maldives, however, further sampling sessions and a longer study period might yield a better result in the future.

## Figures and Tables

**Figure 3a:**
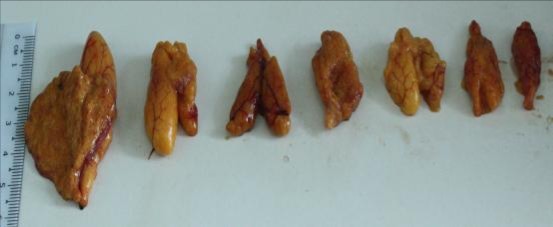
Ovary size variation in *Selar crumenophthalmus*. From right to left, as the ovaries matured their size and development increased.

**Figure 3b:**
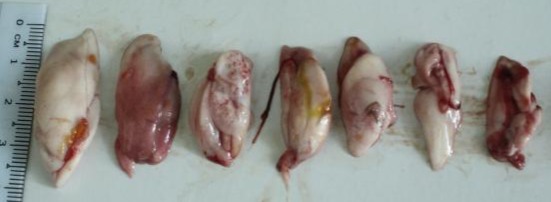
Testis size variation in *Selar crumenophthalmus*. From right to left, as the testis matured their size and development increased.

**Figure 4a:**
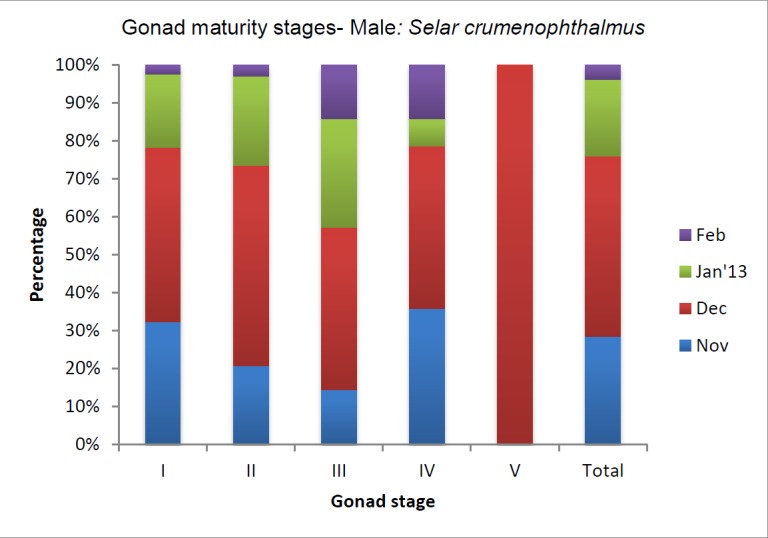
Male gonad maturity stages of *Selar crumenophthalmus*.

**Figure 4b:**
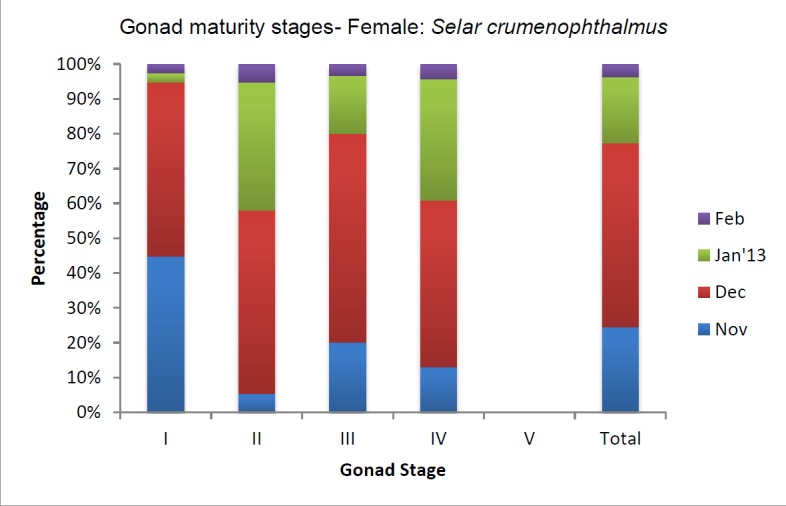
Female gonad maturity stages of *Selar crumenophthalmus.*

**Figure 6a:**
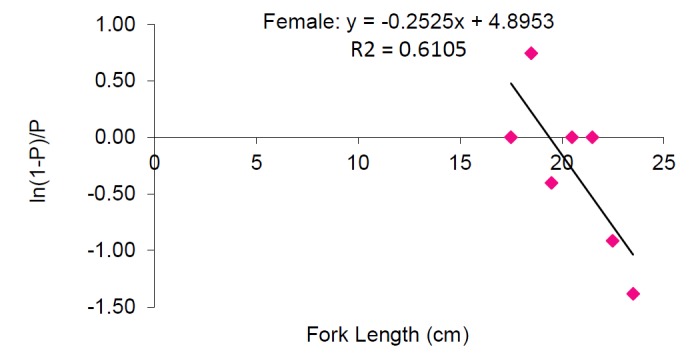
Length at first maturity for female *Selar crumenophthalmus.*

**Figure 6b:**
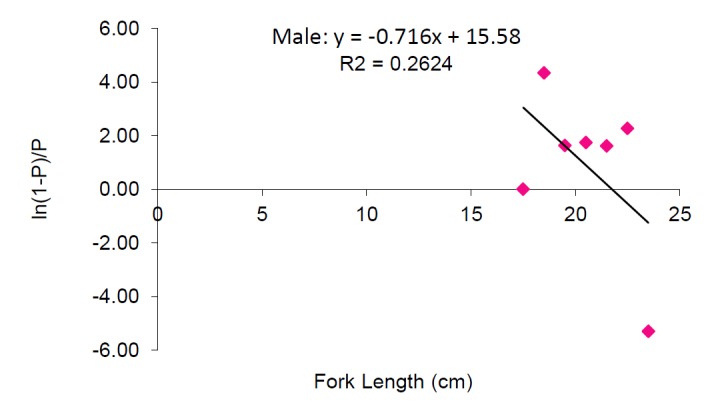
Length at first maturity for male *Selar crumenophthalmus.*

**Figure 7a:**
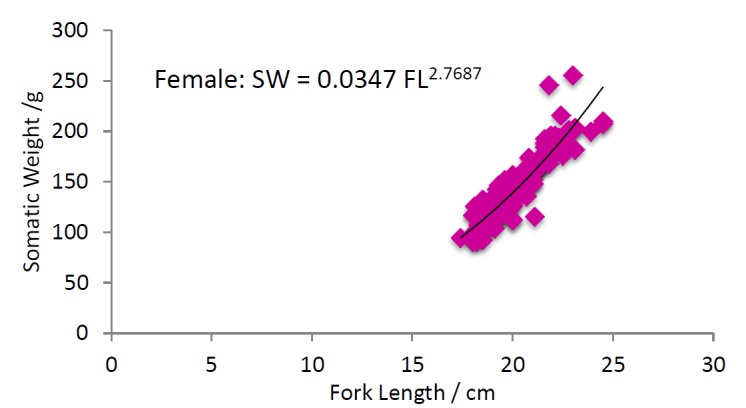
Length-weight relationship of female *Selar crumenophthalmus.*

**Figure 7b:**
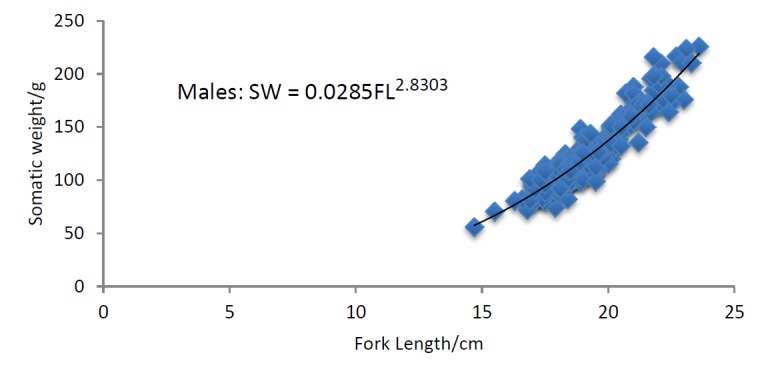
Length-weight relationship of male *Selar crumenophthalmus.*

**Figure 7c:**
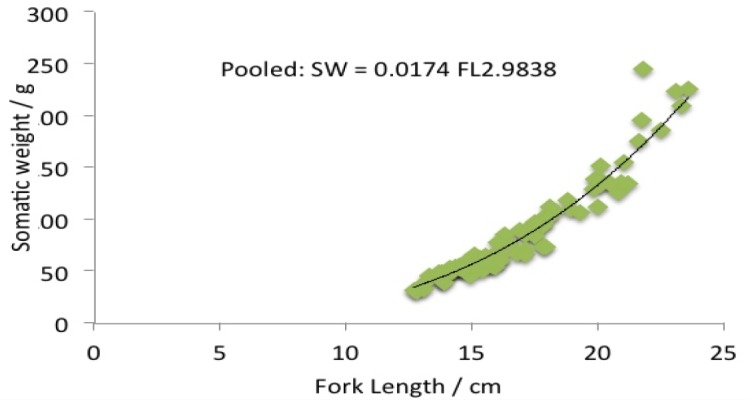
Length-weight relationship of pooled data *Selar crumenophthalmus.*

**Table 1:** The estimated sex ratio values for *Selar crumenophthalmus* in Bangaa Faru.

Sex ratio					

	Male	Female	Immature	M	F
September 2012	67	20	22	1	0.30
October 2012	32	13	92	1	0.41
November 2012	71	28	222	1	0.39
December 2012	125	58	383	1	0.46
January 2013	51	20	365	1	0.39
February 2013	11	4	64	1	0.36

## References

[b1-tlsr-28-2-127] Adam MS (2006). Country review: Maldives. Review of the state of world marine capture fisheries management: Indian Ocean FAO Fisheries Technical Paper.

[b2-tlsr-28-2-127] Adeeb S, Fadzly N, Sah ASRM (2014). Population dynamics of bigeye scad, *Selar crumenophthalmus* in Bangaa Faru, Maldives. Journal of Marine Biology and Oceanography.

[b3-tlsr-28-2-127] Beverton RJH, Holt SJ (1957). On the dynamics of fish population. Fisheries Investment.

[b4-tlsr-28-2-127] Charles A (2005). Observations of crustaceans in the Maldives 1990–2002. Journal of Cetacean Research and Management.

[b5-tlsr-28-2-127] Clarke TA, Privitera LA (1995). Reproductive biology of two hawaiian pelagic carangid fishes, the bigeye scad, *Selar crumenophthalmus*, and the round scad, *Decapturus macarellus*. Bulletin of Marine Science.

[b6-tlsr-28-2-127] Dalzella P, Peñaflor G (1989). The fisheries biology of bigeye scad, *Selar crumenophthalmus (*Bloch) in the Philipines. Asian Fisheries Science.

[b7-tlsr-28-2-127] Dulčič J, Kraljević M (1996). Weight-length relationship for 40 fish species in the Eastern Adriatic (Croation waters). Fisheries Resources.

[b8-tlsr-28-2-127] Froese R (2006). Cube law, condition factor and weight-length relationships: history, meta-analysis and recommendations. Journal of Applied Ichthyology.

[b9-tlsr-28-2-127] Gonzales BJ, Palla HP, Mishina H (2000). Length-weight relationship of five serranids from Palawan Island, Philippines. Naga, the ICLARM Quarterly.

[b10-tlsr-28-2-127] Hafiz A (1990). The biology and growth of bigeye scad (*Selar crumenophthalmus*; Carangidae) in Maldivian waters. Rasain.

[b11-tlsr-28-2-127] HIES (2012). House hold income and expenditure survey. Male’ Maldives.

[b12-tlsr-28-2-127] Holden MJ, Raitt DFS (1974). Manual of fisheries science Part 2: Methods of resource investigation and their application.

[b13-tlsr-28-2-127] Iwai TY, Tamaru CS, Yasukochi L, Alexander S, Yoshimura R, Mitsuyasu M (1996). Natural spawning of captive bigeye scad *Selar crumenophthalmus* in Hawaii. Journal of the World Aquaculture Society.

[b14-tlsr-28-2-127] John C, Benoit Ml, Peter W, Matt PH (2009). Notes on nine biological indicators estimable from trawl surveys with an illustrative assessment for North Sea cod. Aquatic Living Resources.

[b15-tlsr-28-2-127] Joseph MM, Jayaprakash AA (2003). Status of exploited marine fishery resources of India.

[b16-tlsr-28-2-127] Kawamoto PY (1973). Management investigation of the akule or big-eye scad, *Trachurops crumenophthalmus* (Bloch). Unpublished Project Report No. H-4-R.

[b17-tlsr-28-2-127] Kazama TK (1977). The “akule” fishery of Hawaii. South pacific comission. Ninth regional technical meeting of fisheries.

[b18-tlsr-28-2-127] Kipling C (1962). The use of scales of the brown trout (*Salmo trutta L*.) for the back-calculation of growth. l’Exploration de la Mer.

[b19-tlsr-28-2-127] Latheefa A (2004). State of the Environment 2004. Male’ Maldives.

[b20-tlsr-28-2-127] Latheefa A, Shafia A, Shafeega F (2011). State of the Environment 2011.

[b21-tlsr-28-2-127] Le Cren ED (1951). The length weight relationship and seasonal cycle in gonad weight and condition in the perch *(Perca fluviatillis)*. Journal of Animal Ecology.

[b22-tlsr-28-2-127] Lleonart J, Salat J, Torres GJ (2000). Removing allometric effects of body size in morphological analysis. Journal of Theoretical Biology.

[b23-tlsr-28-2-127] Mablouke C, Kolasinski J, Potier M, Cuvillier A, Potin G, Bigot L, Frouin P, Jaquemet S (2013). Feeding habits and food partitioning between three commercial fish associated with artificial reefs in a tropical coastal environment. African Journal of Marine Science.

[b24-tlsr-28-2-127] Mansor MI, Syed A, Abdul Hamid Y (1996). Population structure of small pelagic fishes off the east coast of peninsular Malaysia.

[b25-tlsr-28-2-127] Morgan MJ (2008). Integrating reproductive biology into scientific advice for fisheries management. Journal of Northwest Atlantic Fisheries Science.

[b26-tlsr-28-2-127] Nelson FF, Aloísio SB, Milani PCC (2009). Estimating size at first maturity (L_50_) from Gonadossomatic Index (GSI) data. Neotropical Ichthyology.

[b27-tlsr-28-2-127] Panda D, Jaiswar AK, Sarkar SD, Chakraborty SK (2015). Growth, mortality and exploitation of bigeye scad, Selar crumenophthalmus off Mumbai, north-west coast of India. Journal of the Marine Biological Association of the United Kingdom.

[b28-tlsr-28-2-127] Podosinnikov A (1990). Infrmation on the development of scads in the Gulf of Aden. Journal of Ichthyology.

[b29-tlsr-28-2-127] Ricker WE (1958). Handbook of computation for biological studies of fish populations.

[b30-tlsr-28-2-127] Roos D, Olivier R, François C (2007). Notes on the biology of bigeye scad, Selar crumenophthalmus (Carangidae) around Reunion Island, Southwest Indian Ocean. Scientia Marina.

[b31-tlsr-28-2-127] Rounsefell GA, Everhart WH (1953). Fishery science: its methods and applications.

[b32-tlsr-28-2-127] Roux O, Conand F (2000). Feeding habits of the bigeye scad, *Selar crumenophthalmus* (Carangidae), in La Réunion island waters (south-western indian ocean). Cybium.

[b33-tlsr-28-2-127] Rumpet R, Awang D, Musel J, Biusing R (1997). Distribution, abundance and biological studies of economically important fishes in the South China Sea, Area II: Sarawak, Sabah and Brunei Darussalam Waters. Fisheries Bulletin.

[b34-tlsr-28-2-127] Sadhamoto B, Atmadja SB (1985). On the growth of some small pelagic fish in the Jawa sea. Perikan Laut.

[b35-tlsr-28-2-127] Shameen A, Dutt S (1984). A note on sexual dimorphism in carangid fishes. Mahasagar.

[b36-tlsr-28-2-127] Solah M (2007). A bioeconomic analysis of Maldivian kipjack tuna fishery. Unpublished Master Thesis.

[b37-tlsr-28-2-127] Silberberg KR, Laidig TE, Adams PB, Albin D (2001). Analysis of maturity in lingcod, Ophiodon elongatus. California Fish and Game.

[b38-tlsr-28-2-127] Vicentini RN, Araujo FG (2003). Sex ratio and size structure of *Micropogonias furnieri* (Desmarest, 1823) (Perciformes, Sciaenidae) in Sepetiba bay, Rio de Janeiro, Brazil. Brazil Journal of Biology.

[b39-tlsr-28-2-127] Weng K, Sibert JR (2000). Analysis of the Fisheries for two pelagic carangids in Hawaii.

